# Spatial patterns of multidrug resistant tuberculosis and relationships to socio-economic, demographic and household factors in northwest Ethiopia

**DOI:** 10.1371/journal.pone.0171800

**Published:** 2017-02-09

**Authors:** Kefyalew Addis Alene, Kerri Viney, Emma S. McBryde, Archie C. A. Clements

**Affiliations:** 1 Research School of Population Health, College of Medicine, Biology and Environment, The Australian National University, Canberra, Australian Capital Territory, Australia; 2 Institute of Public Health, College of Medicine and Health Sciences, University of Gondar, Gondar, Ethiopia; 3 Centre for Population Health, Burnet Institute, Melbourne, Victoria, Australia; 4 Department of Medicine, The University of Melbourne, Parkville, Victoria, Australia; 5 Australian Institute of Tropical Health and Medicine, James Cook University, Townsville, Queensland, Australia; Institut de Pharmacologie et de Biologie Structurale, FRANCE

## Abstract

**Background:**

Understanding the geographical distribution of multidrug-resistant tuberculosis (MDR-TB) in high TB burden countries such as Ethiopia is crucial for effective control of TB epidemics in these countries, and thus globally. We present the first spatial analysis of multidrug resistant tuberculosis, and its relationship to socio-economic, demographic and household factors in northwest Ethiopia.

**Methods:**

An ecological study was conducted using data on patients diagnosed with MDR-TB at the University of Gondar Hospital MDR-TB treatment centre, for the period 2010 to 2015. District level population data were extracted from the Ethiopia National and Regional Census Report. Spatial autocorrelation was explored using Moran’s I statistic, Local Indicators of Spatial Association (LISA), and the Getis-Ord statistics. A multivariate Poisson regression model was developed with a conditional autoregressive (CAR) prior structure, and with posterior parameters estimated using a Bayesian Markov chain Monte Carlo (MCMC) simulation approach with Gibbs sampling, in WinBUGS.

**Results:**

A total of 264 MDR-TB patients were included in the analysis. The overall crude incidence rate of MDR-TB for the six-year period was 3.0 cases per 100,000 population. The highest incidence rate was observed in Metema (21 cases per 100,000 population) and Humera (18 cases per 100,000 population) districts; whereas nine districts had zero cases. Spatial clustering of MDR-TB was observed in districts located in the Ethiopia-Sudan and Ethiopia-Eritrea border regions, where large numbers of seasonal migrants live. Spatial clustering of MDR-TB was positively associated with urbanization (RR: 1.02; 95%CI: 1.01, 1.04) and the percentage of men (RR: 1.58; 95% CI: 1.26, 1.99) in the districts; after accounting for these factors there was no residual spatial clustering.

**Conclusion:**

Spatial clustering of MDR-TB, fully explained by demographic factors (urbanization and percent male), was detected in the border regions of northwest Ethiopia, in locations where seasonal migrants live and work. Cross-border initiatives including options for mobile TB treatment and follow up are important for the effective control of MDR-TB in the region.

## Introduction

Tuberculosis (TB), a disease that has killed approximately 2 billion people over the last 200 years, remains a threat to humankind[1]. It disproportionally affects those living in low and middle income countries, and within countries, people from low socio-economic groups [1–3]. The most recent global TB report estimated that there were 10.4 million new cases globally (equivalent to an incidence rate of 142 cases per 100,000 population) and 1.4 million deaths in 2015[4]. The geographic distribution of the disease varies across the globe, as well as within countries, with poverty and a number of other risk factors being strong predictors of incidence [5–7]. The continent of Africa reports particularly high incidence rates; it accounts for 26% of all TB cases in the world and the highest reported incidence rate of 275 cases per 100,000 population [4]. Approximately 87% of the global TB burden is reported from 30 high TB burden countries [4, 8]. Ethiopia is one of these 30 high-burden countries and has been classified as having high burdens of TB, multidrug resistant TB (MDR-TB) and TB-HIV co-infection [4, 8]. The country is striving to reduce the magnitude of the disease in line with the objectives of the global End TB Strategy[9].

Multidrug-resistant TB is defined as TB that is resistant to at least isoniazid and rifampicin [10]. The emergence of MDR-TB has posed an additional challenge for global and national TB control efforts. Globally, in 2015, there were an estimated 481,400 new cases of MDR-TB, and approximately 250 000 deaths from MDR-TB[4]. According to the 2016 WHO global TB report, in Ethiopia an estimated 2.7% and 14% of new and previously treated TB cases had MDR-TB[11]. Among notified pulmonary TB cases, more than 3 300 MDR-TB patients were estimated to occur in 2015 (equivalent to an incidence rate of 3.4 cases per 100,000 population). The MDR-TB case-detection rate is very low; less than a quarter of the estimated 3 300 MDR-TB patients were identified in 2015. However, it is improving and the reported number of patients with MDR-TB has increased from 140 in 2010 to 597 in 2015[11, 12]. The reasons behind the emergence of MDR-TB are multi-factoral, and are associated with socio-economic, demographic, cultural, behavioural, clinical and environmental factors. Previous spatial studies on MDR-TB conducted in Peru [13–17], Moldova [18] and Georgia [19] have shown that MDR-TB is clustered in specific geographical areas and clusters are associated with location, socio-economic status and population density [20, 21].

The identification of areas where MDR-TB is concentrated could allow policy makers to implement targeted interventions aimed at prevention and management. This might be particularly important in resource-constrained settings and in high MDR-TB burden countries. Targeted interventions may be necessary in countries that find it impossible to provide universal MDR-TB services across the country as the diagnosis and treatment of MDR-TB is expensive. The Ethiopian 2013 national TB report indicated that a significant proportion of MDR-TB patients did not get the opportunity to access MDR-TB treatment due to the scarcity of treatment centers and inadequate diagnostic facilities [22].

The use of geographical information systems (GIS) and spatial analysis to identify hot spots of disease [23], may be helpful to identify the geographical patterns and ecological predictors of MDR-TB in high TB burden countries such as Ethiopia. However, there are very few studies in Africa that have assess the spatial distribution of TB. Hence, the aims of this study were: 1) to determine the spatial distribution of MDR-TB in northwest Ethiopia using a six-year cohort; and 2) to identify district-level socio-economic, demographic, household and environmental variables associated with the spatial distribution of MDR-TB in this region.

## Methods

### Study design and area

**Fig 1:**
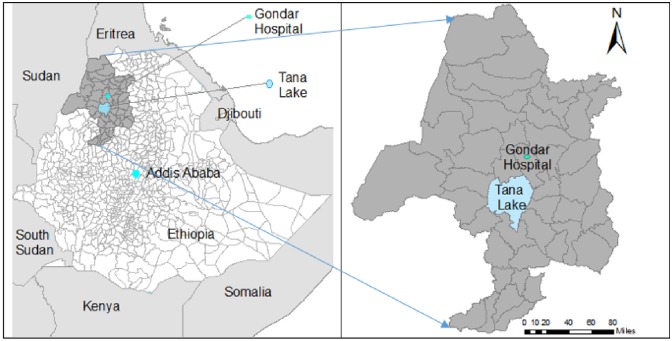
Map of the study area, northwest Ethiopia.

This was an ecological study, using MDR-TB data from northwest Ethiopia aggregated at the district level. Northwest Ethiopia has a variety of geographical features, including the highest mountain and the largest lake in the country, and a geographical boundary with both Sudan and Eritrea (Fig 1).

More than 8 million people live in the area, in 45 different districts and towns; approximately 85% of the population lives in rural areas [24]. The University of Gondar Hospital is one of the oldest referral hospitals in the area and the only hospital that provides MDR-TB services in northwest Ethiopia. Individuals who are living in northwest Ethiopia and who developed resistance to first line anti-TB drugs or who are suspected of having MDR-TB are referred to University of Gondar Hospital for diagnosis and treatment.

While TB patients can be diagnosed using sputum smear microscopy and treated with first line anti-TB drugs at the local level, MDR-TB patients are diagnosed (based on the laboratory results obtained from the regional or national laboratory centre) and treated at Gondar University Hospital. In the Ethiopian context, the national TB guideline recommends that drug-susceptibility testing (DST) is used for high risk groups who are at increased risk of having drug resistant TB. High risk groups are identified based on patient history, such as: previous exposure to anti-TB treatment (i.e. a treatment outcome of failure, treatment after relapse, treatment after loss to follow up and previous treatment with an unknown treatment outcome), exposure to a known MDR-TB case, co-morbid conditions such as HIV/AIDS and other conditions associated with mal-absorption[22, 25]. Patients suspected of having MDR-TB are referred to University of Gondar Hospital for further diagnosis and treatment. The hospital staff collect a sputum sample from the patient and send it to the national or regional laboratory for confirmation of the diagnosis. Phenotypic DST, line probe assay (i.e Geno Type MTBDRplus V.2.o, HAIN Life, Science, Nehren, Germanuny) and GeneXpert are performed at national and regional reference laboratories to identify MDR-TB cases. Patients diagnosed with MDR-TB are treated at Gondar University Hospital free of charge. Details about the treatment regimen used have been described elsewhere[26].

### Study population

The study population included all MDR-TB cases registered and living in the catchment area of the University of Gondar Hospital MDR-TB treatment centre (45 contiguous districts in northwest Ethiopia), registered between the years 2010 and 2015. Patients whose residence lay outside of the hospital’s catchment area (i.e. from adjacent regions and districts) were excluded from the study.

### Data source and variables of the study

#### Dependent variable

The number of MDR-TB patients in each district was the dependent variable. The MDR-TB dataset included individual-level demographic, laboratory and clinical variables. The data were collected by staff from the University of Gondar MDR-TB treatment centre. These MDR-TB data were aggregated to the district level to obtain the number of MDR-TB patients in each district for each year.

#### Independent variables

District-level ecological data were included as independent variables. A number of variables were extracted from the Ethiopian national and regional census report[24, 27]. These variables included demographic factors (i.e. percentage of male and female, percentage of people living in rural and urban communities); socioeconomic variables (i.e. illiteracy rate, economic inactivity and the unemployment rate); housing conditions (i.e. the average number of households per housing unit and the average number of rooms per house); measures of indoor air pollution (i.e. percentage of houses with traditional kitchens and using charcoal, firewood and dung for cooking); and migration variables (i.e. the percentage of all migrant types in the area and the percentage of new migrants (less than 5 years) in the area). The term economic inactivity, according to the Ethiopian Population and Housing Census report, refers to the proportion of people aged ten years and above who were neither engaged nor available to be engaged in the production of economic goods and services during a given reference period [24].

A polygon shapefiles for Ethiopia’s administrative boundaries at the district level was obtained from the Open Africa website [28]. The boundaries of the districts required for the study were selected. All of the extracted data were linked and georeferenced with the district polygons using the GIS, ArcGIS 10.1.3 [29]. The population density of each district was calculated using ArcGIS by dividing the total population in each district by the area of that district in square kilometres.

### Data analysis

#### MDR-TB incidence rate

The overall crude incidence rate of MDR-TB was calculated by taking the total number of MDR-TB cases reported in the six-year study period as the numerator and the mid-point total population during the same time period as the denominator. The sex and residence adjusted standardized morbidity ratio (SMR) was calculated for each district using the formula: Y_i_ = [O_i_/E_i_]; where Y is the SMR in district i, O is the observed number of MDR-TB cases in the district and E is the expected number of MDR-TB cases in the district across the study period. The expected number of MDR-TB cases for each district was calculated by multiplying the mid-point population of each district by the overall crude MDR-TB incidence rate for the study area and period.

#### Spatial autocorrelation analysis

Spatial autocorrelation was explored at a global scale using Moran’s I statistic and at a local scale using Local indicators of spatial association (LISA), estimated using the Anselin Local Moran’s I statistic, and the Getis-Ord statistic. The global Moran’s I statistic was used to assess the presence, strength and direction of spatial autocorrelation over the whole study area and to test the assumption of spatial independence in the implementation of the spatial pattern analysis. The LISA and the Getis-Ord statistics were used to detect local clustering of MDR-TB and to identify the locations of hot-spots. These analyses were conducted using tools provided in ArcGIS.

#### Non-spatial Poisson regression analysis

Because the dependent variable (i.e. the number of MDR-TB cases in each district) was a count variable and a rare disease, we assumed that it followed a Poisson distribution. Univariate and multivariate Poisson regression models were initially computed using STATA version 14 software (StataCorp. 2015. Stata Statistical Software: Release 14. College Station, TX: StataCorp LP), by taking the number of MDR-TB cases recorded by districts during 2010–2015 as the dependent variable and all other variables as independent variables. Those independent variables that had a p-value less than 0.2 in the univariate Poisson regression model were fitted to the final multivariate Poisson regression model and were considered for further spatial analysis. All significant variables were tested for multi-collinearity and those variables with a variance inflation factor (VIF) greater than 6 were excluded from the final model.

#### Spatial Poisson regression analysis

We constructed three different Bayesian spatial models using WinBUGS version 1.4.3 software (Medical Research Council Biostatistics Unit, Cambridge, United Kingdom). In the first model (Model I), we assumed that spatial autocorrelation was not present in the relative risk of MDR-TB and a model without a spatial component was computed. All of the covariates selected above in the non-spatial multivariate Poisson regression analysis were incorporated as fixed effects, and unstructured random effects for districts were included in the model. The assumptions of this model were that: 1) the number of MDR-TB cases that occurred in one district were independent of the number of MDR-TB cases in the other districts, after accounting for the covariates; and 2) the variance was homogeneous across the study area. To handle the possible spatial dependency of observations and the violation of homogeneity of variance within each district due to the spatial nature of the data, a second model (Model II) containing spatially structured random effects was constructed using a Bayesian smoothing conditional autoregressive (CAR) model for the random effects.

Finally, a third model (Model III), a convolution model, containing the covariates and both the unstructured and spatially structured random effects, was constructed. This model assumed that the observed number of MDR-TB cases (Y) for i^th^ district followed a Poisson distribution with a mean (*μ*):

$$Y_{i}\sim \text{Poisson}(\mu_{i});$$

$$\text{Log }(\mu_{i})=\text{Log}(E_{i})+\theta_{i}$$

$$\theta i=\alpha +\beta_{li}\;X_{\;li}+\ldots ..\beta_{ni}\;X_{\;ni}+U_{i}+V_{i}$$

Where *E*_*i*_ is the sex and residence-adjusted expected number of MDR-TB cases in district *i*; *θ*_*i*_ is the log relative risk (RR) determined by the intercept (*α*), the coefficients of the covariates (*β*_*1i*_ … *β*_*ni*_), the unstructured random effects (*U*_*i*_) and the spatially structured random effects (*V*_*i*_). The spatially structured random effects (*V*_*i*_) were computed using a CAR prior structure, which is defined using an adjacency matrix to determine the spatial relationships between districts. The adjacency matrix for each district was generated using ArcGIS. A weight of 1 was given if two districts were neighbouring and a weight of 0 was given if two districts were not neighbouring. Two districts were considered to be neighbouring if they shared the same edges or corners (i.e. queen contiguity). Prior probability distributions for the coefficients (*β*) were assumed to have normal distributions with a mean = 0 and a precision (i.e. inverse of variance) = 1 x 10^−6^. For the intercept (*α*) flat prior distributions was used (i.e. a non-informative, improper prior with bounds - ∞ and +∞). The unstructured random effects (*U*_*i*_) and spatially structured random effects (*V*_*i*_) were assumed to have a mean of zero and a precision (inverse of variance) of 1/*σ*_*u*_^*2*^ and 1/*σ*_*v*_^*2*^ respectively. The priors for the precision of the unstructured and spatially structured random effects were assigned a non-informative gamma distribution with a shape and scale parameters of 0.001 (S1 Table).

The posterior parameters were estimated from the prior and data likelihood information using a Bayesian Markov Chain Monte Carlo (MCMC) simulation approach with Gibbs sampling, employed by WinBUGS. After an initial burn-in of 1,000 iterations, the models were run subsequently for 1,000,000 iterations. For all models (as evidenced from visual inspection of posterior kernel densities and history plots), convergence occurred within the first 10,000 iterations. One hundred thousand values from the posterior distribution of each parameter were stored for summary measures such as the posterior mean, standard deviation and the 95% credible interval (CrI). The Deviance Information Criterion (DIC) was also stored for model selection, where a lower DIC indicates a preferred model.

### Ethical clearance

Ethical clearance was obtained from the Institutional Review Board of the University of Gondar and from the Australian National University Human Research Ethics Committee (protocol number 2016/219). Further, the University of Gondar Hospital provided permission to access the data. As this study used secondary data, informed consent was not obtained from each of the study participants.

## Results

### Descriptive analysis

Among a total of 282 MDR-TB patients notified during the six year period in northwest Ethiopia, 17 (6.0%) patients resided out of the study area and were therefore excluded from the analysis. Of the 264 patients who were included, 159 (60.2%) were male and 115 (56.4%) were from urban locations. The highest number of MDR-TB cases were reported in the two highly populated and urban areas of the region, namely Gondar (60 cases; 23.0%), the place where the MDR-TB treatment center is located and Bahirdar (31 cases; 12.0%), the capital city of the region. Nine districts, many of them located far away from the treatment centre, did not report any MDR-TB. The overall crude incidence rate of MDR-TB for the six year period was 3.0 cases per 100,000 population, ranging from 0 to 21 cases per 100,000 population, per district.

**Fig 2:**
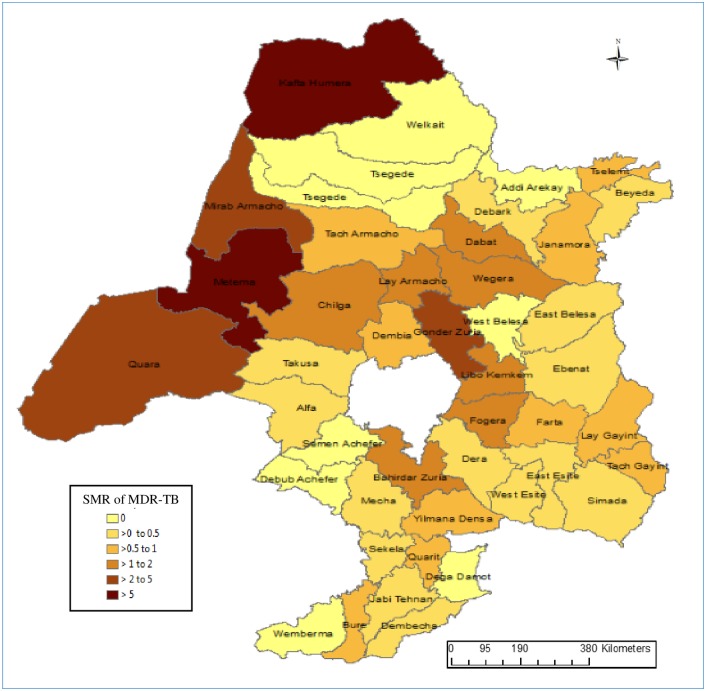
Choropleth map showing the geographical distribution of multidrug-resistant tuberculosis standardized morbidity ratios across each district in the northwest Ethiopia, 2010 to 2015.

We observed strong spatial variation in the MDR-TB incidence rate across the districts. The map in Fig 2 shows the spatial distribution of the MDR-TB SMR in northwest Ethiopia at the district level. The highest SMR was observed in Metema (7.0) and Humera (6.1) districts, both located in the Ethiopia-Sudan and Ethiopia-Eritrea border regions of the country (Fig 2).

### Spatial distribution of MDR-TB

**Fig 3:**
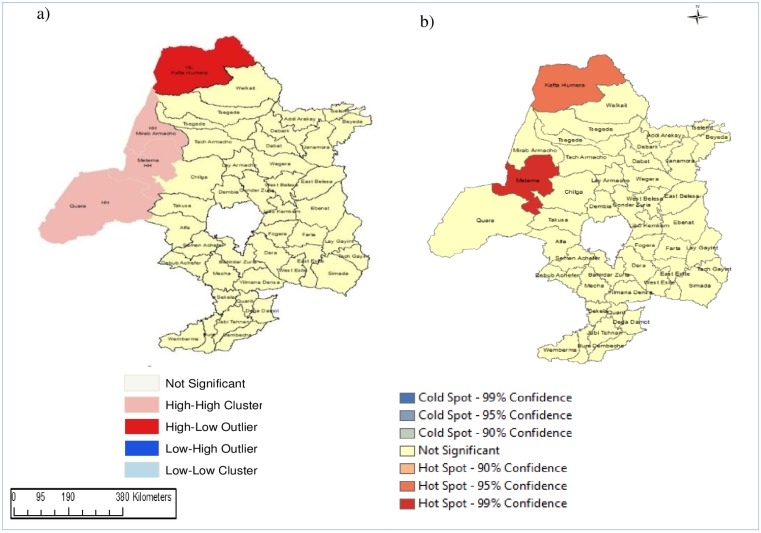
Spatial clustering of multidrug-resistant tuberculosis incidence in northwest Ethiopia, 2010 to 2015, based on: a) Local indicators of spatial association using Anselin Local Moran’s I statistic; and b) and the Getis-Ord Gi* statistic.

The global Moran’s index statistic for the MDR-TB incidence rate per 100,000 population was 0.14 (p-value = 0.04), indicating the presence of significant, positive spatial autocorrelation in MDR-TB incidence rate over the whole study area. In the LISA analysis, districts such as Quara, Metema and Mirab Armacho (all located in the border regions) showed a high-high type of relationship, meaning that these districts had a high incidence of MDR-TB and the surrounding districts had also had high MDR-TB incidence (Fig 3a). Humera district, the other district which is also found in the border region of the country, had a high-low type of relationship which indicated that there was a high incidence of MDR-TB in this district, surrounded by districts with low incidence of MDR-TB. Using the Getis-Ord statistic (Fig 3b), we observed that Metema and Humera were hot spots at 99% and 95% confidence levels, respectively. There were no statistically significant relationships for all other districts (Fig 3).

### Factors associated with MDR-TB clusters

The multivariable Poisson regression results are presented in Table 1. At the area level, the incidence of MDR-TB per 100,000 population was positively associated with population density per square kilometer (RR: 1.01; 95% CI: 1.00, 1.01), the proportion of the population who were economically inactive (RR: 1.05; 95% CI: 1.03, 1.07), the proportion of people living in urban areas (RR: 1.02; 95%CI: 1.01, 1.04) and the proportion of males in the population (RR: 1.58; 95% CI: 1.26, 1.99).

**Table 1:** A multivariable fixed effects Poisson regression model of socio-economic and demographic factors influencing district-level incidence of multidrug resistant tuberculosis per 100,000 population in northwest Ethiopia, 2010 to 2015.

Variables	Coefficients	Relative risk (95%CI*)	P-value
Population density per square kilometre	0.01	1.01 (1.00, 1.01)	0.001
Economically inactive population (%)	0.05	1.05 (1.03, 1.07)	<0.001
Migrant population (%)	0.001	1.00 (0.99, 1.01)	0.887
Male population (%)	0.46	1.58 (1.26, 1.99)	0.01
Urban residence (%)	0.02	1.02 (1.01, 1.04)	0.03

*Confidence interval.

As indicated by the low DIC value in Table 2, the model without a spatial component had a better fit than the models containing spatially structured random effects. This suggested that despite the presence of spatial autocorrelation for the dependant variables in the exploratory phase of the study, and as evidenced by the global and local Moran’s I statistic, the inclusion of covariates resulted in no residual spatial autocorrelation, and thus the CAR random effects models were redundant. In other words, the covariates included in the models explained the spatial autocorrelation and spatial clustering evident in the MDR-TB data.

**Table 2:** Poisson regression model for the association of socio-economic, demographic, housing condition and spatially structured random effect at the district level with cases of multidrug-resistant tuberculosis in northwest Ethiopia, 2010 to 2015.

	Model I: unstructured		Model II: structured		Model III: structured & unstructured	
Variable	Coefficient, posterior mean (95% CrI^a^)	RR^b^, Posterior Mean (95% CrI^a^)	Coefficient, posterior mean (95% CrI^a^)	RR^b^, Posterior Mean (95% CrI^a^)	Coefficient, posterior mean (95% CrI^a^)	RR^b^, Posterior Mean (95% CrI^a^)
α (Intercept)	-1.2 (-1.67,-0.78)		-1.20 (-1.52,-0.90)		-1.21(-1.66, -0.81)	
Male (%)	**0.29 (0.05, 0.54)**	**1.35 (1.05, 1.71)**	**1.16 (0.88, 1.44)**	**3.22 (2.41, 4.23)**	**0.29 (0.05, 0.54)**	**1.35 (1.05, 1.72)**
Urban residence (%)	**1.16 (0.88, 1.44)**	**3.22 (2.40, 4.23)**	**0.29 (0.05, 0.54)**	**1.35 (1.05, 1.72)**	**1.16 (0.88, 1.44)**	**3.21 (2.40, 4.22)**
Population density^d^	0.19 (-0.23, 0.62)	1.24 (0.79, 1.87)	0.18 (-0.35, 0.71)	1.24 (0.70, 2.04)	0.19 (-0.28, 0.66)	1.24 (0.76, 1.94)
Migrants (%)	-0.10 (-0.55, 0.34)	0.93 (0.58, 1.41)	-0.26 (-0.72, 0.20)	0.79 (0.49, 1.22)	-0.14 (-0.61, 0.32)	0.90 (0.54, 1.38)
Economically inactive (%)	-0.15 (-0.53, 0.23)	0.87 (0.59, 1.26)	-0.17 (-0.58, 0.26)	0.87 (0.56, 1.29)	-0.15 (-0.53, 0.27)	0.88 (0.59, 1.31)
Heterogeneity						
Unstructured variance	1.06 (0.57, 2.29)		-		0.03 (0.002, 2.18)	
Structured variance	-		3.70 (1.96, 8.33)		0.008 (0.001, 5.56)	
DIC^c^	561.7		568.5		564.1	

^a^Credible interval,

^b^relative risk,

^c^deviance information criterion,

^d^population density per square kilometer

**Fig 4:**
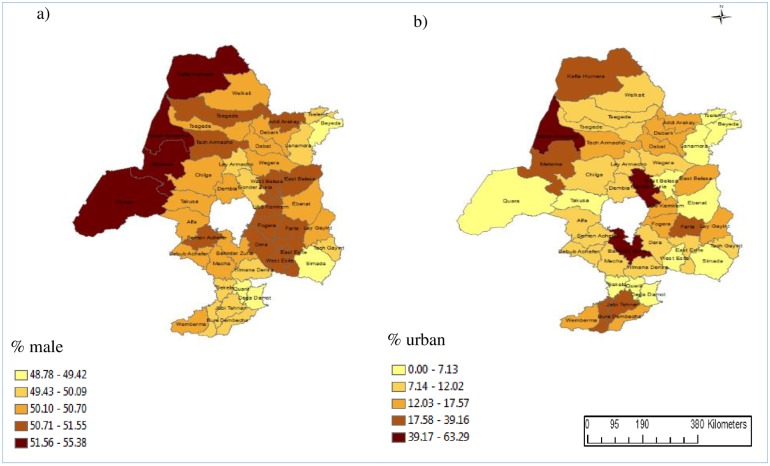
Choropleth maps showing the geographical distribution of the percentage of the district (a) who are male and (b) who live in urban communities across northwest Ethiopia, 2010 to 2015.

The geographical distribution of MDR-TB in the districts was positively associated with the percentage of the population that was male and the percentage of the population living in urban locations. The other variables included in the models were not significantly associated with MDR-TB incidence. Maps of the percentage of the population who were male and who were living in urban locations are provided in Fig 4, demonstrating that the spatial distribution of the disease and these predictors were similar.

## Discussion

We found that MDR-TB was geographically clustered in the north-west border districts of northwest Ethiopia. We also found several characteristics of the districts that were associated with higher rates of MDR-TB including the proportion of males in the population, urbanisation and population density. Of interest, spatial clustering was not apparent once these district characteristics were taken into account, suggesting that the proportion of males and urbanisation explained the spatial distribution of MDR-TB in this region of Ethiopia.

Spatial clusters were detected in Metema and Humera districts, both of which are located in the Ethiopia-Sudan and Ethiopia-Eritrea border regions of the country. These districts are located far from referral hospitals and contain a high number of seasonal migrants (mostly male) due to the presence of agricultural investments in these areas. The detection of clusters of MDR-TB in agricultural investment areas and in the border regions of the country may mean that MDR-TB is poorly controlled in these itinerant and hard to reach populations. This highlights the risk of cross-border transmission of MDR-TB in Ethiopia, Eritrea and Sudan, particularly in predominantly male migrant populations. Therefore, we suggest that mobile TB and MDR-TB services should be considered to address the issue of TB among the seasonal migrant worker population in the border regions.

In previous studies, similar cross-border problems of MDR-TB in east Africa along the Somalia-Kenya border [30] and in Mongolia along the Trans-Siberian Railway line[31] have been reported. This reinforces the need for cross-border collaboration for the effective control of MDR-TB [32].

The majority of MDR-TB patients in our study were male. Previous studies conducted in Ethiopia have also reported that MDR-TB is more common among males [33, 34]. This could be related to the convergence of other risk factors for TB, such as cigarette smoking and the need for a long duration of treatment (noting that, as males are more likely to travel from place to place in Ethiopia, particularly as seasonal migrants, they may have a higher likelihood of being infected with TB or experiencing treatment interruptions) [35, 36]. Poor compliance with anti-TB drugs has previously been documented among seasonal migrants [37] and MDR-TB is more prevalent among people who have not completed a course of TB treatment [38].

We also found that a higher risk of MDR-TB was associated with urbanisation. Another study conducted in Georgia found similar findings, where living in the capital city was a risk factor for MDR-TB [19]. This may be due to the fact that transmission of MDR-TB may be more common in urban settings due to overcrowding and higher population density [39].

The clustering of MDR-TB in the community is due to the transmission of drug resistant strains (i.e. primary resistance) [40, 41], or acquisition of drug resistance as a result of poor first line anti-TB treatment[42]. Further studies are needed to identify whether the spatial clustering of MDR-TB in the districts is due to primary transmission of drug resistant strains as a result of high population density and urbanization or due to poor first line TB treatment, weak health care services or behavioural factors.

Percent of the population who were migrants was not statistically significant in the model despite the fact that clusters of cases were observed in the area where seasonal migrants live and work. This might be because we used the percentage of population who were immigrants to the district within the last five years of the census as “migrants”, and these people are different from “seasonal migrants” who are living in the cross-border areas of the country for short periods of time, usually for less than 3 months. The majority of seasonal migrants in the border area who come from different parts of the country were not counted in the census report as a migrant due to the fact that a six month cut off was used. This might result in the percentage of migrants in the area being under-estimated, impacting on the ability to detect a significant effect in the models.

This study has some limitations. First, whilst the level of analysis was by district, it might be preferable to investigate even smaller geographical units such as kebeles. However, reliable and comprehensive information at this level is simply not available for both the dependent and independent variables. Our study may only allow us to reach conclusions at the district level, and we cannot generalize the finding to the individual or kebele levels.

Second, since our study is based on reported cases of MDR-TB, it may not reflect the exact magnitude of the disease, especially in rural settings. Despite the fact that Ethiopia is classified as a high MDR-TB burden country, the MDR-TB incidence rate in northwest Ethiopia for the six year period is approximately 3 cases per 100,000 population, which is much lower than both the national (3.4 cases per 100,000 population per year) and global (6.6 cases per 100,000 population per year) estimated MDR-TB incidence rate[11]. This low MDR-TB incidence may indicated a low MDR-TB detection rate. However, since the establishment of the MDR-TB treatment center at University of Gondar hospital in 2010, all MDR-TB cases in northwest Ethiopia are admitted to the hospital and are also reported to the hospital surveillance system. This has contributed to more reliable data on MDR-TB in the catchment area of the hospital.

## Conclusion

Spatial clustering of MDR-TB was detected in the border regions of Ethiopia in Metema and Humera districts, areas with large numbers of seasonal migrants living far from referral hospitals. A mobile treatment approach for seasonal migrants, and collaborative focused interventions at cross-border areas might be important to consider for the effective control of MDR-TB in this part of Ethiopia.

## Supporting information

S1 Table The non-spatial, spatial, and both the spatial and non-spatial models constructed using WinGUGS to estimate the posterior parameter. (DOCX)
