# Supplementary material for: Spatial patterns of multidrug resistant tuberculosis and relationships to socio-economic, demographic and household factors in northwest Ethiopia
Source: PLoS One. 2017 Feb 9;12(2):e0171800. doi: 10.1371/journal.pone.0171800 (PMC5300134; doi:10.1371/journal.pone.0171800)
Supplement: S1 Table — (DOCX) [file pone.0171800.s001.docx]

**Supporting information 1**

**Table:** The non-spatial, spatial, and both the spatial and non-spatial models constructed using WinGUGS to estimate the posterior parameter.

| Model I: Non-spatial model | Model II: Spatial model | Model III: both spatial & non-spatial |
| --- | --- | --- |
| *Yi* ~ dPoisson (*µ _i_*);  Log (*µ _i_*) = Log *Ei* + *θi*;  *θi= α + β_1i_ X _1i_ + ….. β_ni_ X _ni_ + U_i_ ;*  *U_i_* ~ dnormal (0.0, *tau*);  Beta (*β_1i_*) ~ dnorm(0.0,1.0E-6);  Alpha (*α*) ~ dnorm(0.0,1.0E-4);  *tau* ~ dgamma(0.001, 0.001); | *Yi* ~ dPoisson (µ _i_);  Log (*µ _i_*) = Log *Ei + θi;*  *θi= α + β_1i_ X _1i_ + ….. β_ni_ X _ni_ + V_i;_*  *V_i_ ~ car*. *Normal* (*adjacencyi , weighti, numberi, tau.v*);  Beta (*β_1i_*) ~ dnorm(0.0,1.0E-6);  Alpha (*α*) ~ dflat() ;  *tau.v* ~ dgamma(0.001, 0.001); | *Yi* ~ dPoisson (µ _i_);  Log (*µ _i_*) = Log *Ei + θi*  *θi= α + β_1i_ X _1i_ + ….. β_ni_ X _ni_ + U_i_ + V_i;_*  *U_i_* ~ dnormal (0.0, *tau*);  *V_i_ ~ car. Normal* (*adjacencyi , weighti, numberi, tau.v);*  Beta (*β_1i_*) ~ dnorm(0.0,1.0E-6);  Alpha (*α*) ~ dflat() ;  *tau.u* ~ dgamma(0.001, 0.001) ;  *tau.v* ~ dgamma(0.001, 0.001); |
